# Assessing diastolic function using CMR as an alternative to echocardiography: age- and gender-related normal reference values

**DOI:** 10.1007/s00392-024-02553-9

**Published:** 2024-09-30

**Authors:** Lilly Charlotte Cirener, Hermann Körperich, Peter Barth, Anca Racolta, Misagh Piran, Wolfgang Burchert, Oliver M. Weber, Jan Eckstein

**Affiliations:** 1 Institute for Radiology, Nuclear Medicine and Molecular Imaging, Heart and Diabetes Center North Rhine Westphalia, Ruhr-University of Bochum, Georgstr. 11, 32545 Bad Oeynhausen, Germany; 2 Clinic for Pediatric Cardiology, Center for Congenital Heart Defects, University Hospital RWTH Aachen, Aachen, Germany; 3 Philips Clinical Science, Hamburg, Germany

**Keywords:** Diastolic function, Cardiac magnetic resonance imaging, Pulmonary venous blood flow, Transmitral blood flow, Echocardiography

## Abstract

**Background:**

Impaired diastolic function is associated with a variety of diseases such as myocarditis or dilated cardiomyopathy. Currently, echocardiography is the standard method for assessing diastolic function. Recently, it has been postulated that cardiovascular magnetic resonance (CMR) is an at least equivalent or superior alternative to echocardiography. To assess CMR-based age- and gender-dependent diastolic functional normal reference values, pulmonary venous and transmitral blood-flow parameters were examined in heart-healthy test persons.

**Methods and results:**

Flow-sensitive phase-contrast CMR imaging was performed in the right upper pulmonary vein (RUPV) and at the level of the mitral valve (MV) in 183 healthy subjects (age 10–70 years; 97 women, 86 men). The data was distributed as evenly as possible across all groups. Strong age-dependence was observed for PV S/D; *r* = 0.718, *p* < 0.001 (Pearson product–moment correlation) and for transmitral MV E/A; *ρ* = −0.736, *p* < 0.001 (Spearman’s Rho correlation). Moderate age-dependence was found for PV slope D-wave; *r* = 0.394, *p* < 0.001. Except for MV slope E-wave (male −292 cm/s^2^ interquartile range (IQR) {−338; −243} vs. female −319 ± 82 cm/s^2^; *p* = 0.047), no gender-related differences were observed. In a subgroup (*N* = 100), CMR data were compared with echocardiographic data. Strong correlation was found between CMR and echocardiography for PV S/D; *r* = 0.545, *p* < 0.001 and MV E/A; *ρ* = 0.692, *p* < 0.001.

**Conclusion:**

Diastolic functional parameters change with age, while gender-differences are small. CMR and echocardiography showed similar PV S/D and MV E/A ratios, making CMR a promising alternative for assessing diastolic function.

**Graphical abstract:**

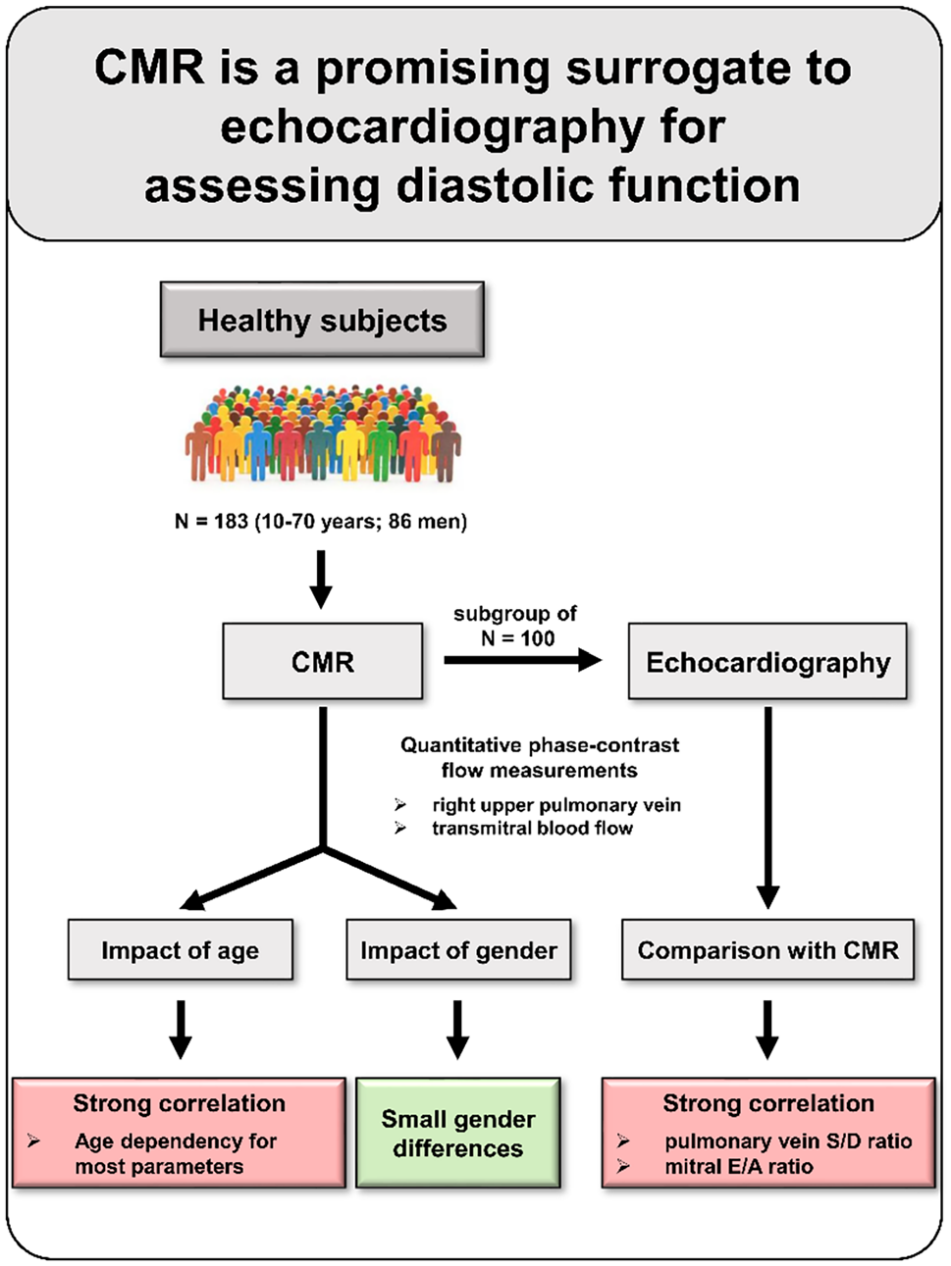

## Background

A reliable and reproducible evaluation of diastolic function is of enormous clinical importance in a variety of internal, especially cardiologic diseases [1], with heart failure being a common example [2]. Echocardiography is currently the standard for assessing diastolic function or dysfunction [3, 4], for which classification guidelines have been established by the American Society of Echocardiography and the European Association for Cardiovascular Imaging [3].

The advantages of echocardiography are the rapid availability, absence of ionizing radiation and portability of the device [5]. However, disadvantages of echocardiography encompass its operator dependence, patient echogenicity and limitations of the acoustic window [6]. To date, cardiovascular magnetic resonance imaging (CMR) is the established gold standard for cardiac functional analysis [7] offering numerous advantages, including examiner independence, high reproducibility, sufficiently highspatial and temporal resolution and radiation-free imaging. However, its role in assessment of diastolic function remains to be clinically determined. Several diastolic functional parameters can be derived by means of CMR such as left ventricular (LV) volume, LV ejection fraction, LV strain, transmitral and pulmonary venous (PV)blood-flow velocity profiles [8–10]. The potential of CMR in visualizing and assessing PV blood flow has been recently described [11]. Simultaneous monitoring of PV and transmitral blood flow characteristics is expected to provide valuable information for the assessment of diastolic dysfunction [12, 13]. However, the rapid expansion of CMR functional analyses, emphasizes the necessity of establishing normal reference values for diastolic function for which literature reporting to date remains scarce.

In diastolic dysfunction the left ventricle becomes stiffer and elasticity decreases, resulting in impaired relaxation and filling of the left ventricle [14, 15]. Diastolic dysfunction is a cardiovascular risk factor and is associated with increased mortality [16, 17]. In routine clinical echocardiography, the following parameters are quantified: E wave, E/A ratio, septal/lateral e′, E/e′, indexed left atrial stroke volume (LA SV_i_) and peak velocities of tricuspid regurgitation [3, 18] as well as the PV S, D and A waves and their corresponding ratios. In addition, to assess the pressure conditions in the left ventricle, it is useful to determine the E/é ratio and septal/lateral é [19].

The study aims to comprehensively evaluate diastolic function employing standard CMR techniques routinely applied in clinical practice. To accomplish this, a systematic analysis of age- and gender-dependent PV and transmitral blood-flow parameters was performed for subsequent determination of diastolic function in a large cohort of healthy individuals. These are of crucial clinical importance as they facilitate discrimination between clinical and subclinical conditions. In addition, a comprehensive investigation of correlations between parameter acquisition from CMR and echocardiography is performed. This study hypothesizes that age- and gender-dependent PV and transmitral blood-flow parameters differ significantly in a healthy cohort and that CMR qualifies as an alternative for echocardiographic assessment of diastolic function.

## Methods

### Study population

This work is a prospective, single-center, cross-sectional study. A total of 208 volunteers were recruited through a public call in the period from September 2017 to December 2020. The study was authorized by the local ethics committee (Ethikkommission der Medizinischen Fakultät der Ruhr-Universität Bochum, Sitz Ostwestfalen, registration number: 2017-238, amendment registration number 2022-924). The current study complies with the principles of the Declaration of Helsinki, seventh revision of 2013.

All study participants or legal guardians signed an informed consent form. All participants completed a questionnaire on their state of health and physical condition in advance to check their suitability for participation in this study. Healthy study participants were selected based on criteria including no cardiovascular disease, no personal or family history of cardiac disease, normal blood pressure, no medications associated with cardiovascular disease, and no associated risk factors or general risk factors for performing CMR. The candidates received a standard information sheet beforehand for both CMR and echocardiography. Routine biventricular (left and right ventricle) CMR function data, including ejection fractions and left ventricular muscle mass, were used to verify that the data were consistent with the normal values published by Kawel-Boehm et al. [20]. Clinical data such as age, gender, weight and height were taken from the questionnaire.

Due to violations of the health criteria, 18 participants had to be excluded from the study. Furthermore, seven participants were excluded due to technical limitations or insufficient image quality.

### Cardiovascular magnetic resonance (CMR)

All study participants received a standard CMR imaging conducted with a multi-transmit 3 T MRI system (Achieva, Philips Healthcare, Best, The Netherlands; Release 5.3.1/5.6.1) with dStream technology at our institution. A built-in posterior coil in the patient support and a dedicated anterior phased-array coil were used for signal reception. All participants were examined in supine position. A vector electrocardiogram was used for triggering and retrospective gating of pulse-sequence acquisitions.

### Assessment of heart function

Standard cine steady-state free-precession (SSFP) acquisitions were applied for 2-chamber, 3-chamber, 4-chamber long axis views and for a short-axis stack covering the left and right ventricles. To assess atrial volumes an additional axial whole-heart cine acquisition was applied. Typical imaging parameters for SSFP acquisitions were repetition time/echo time/flip angle = 2.7 ms/1.35 ms/42°, 45 cardiac phases (interpolated, 32 acquired cardiac phases), and spatial resolution 1.5 × 1.5 × 8 mm^3^. A parallel-imaging factor of 2 using SENSE was applied to expedite sequence acquisition and to keep breath hold times less than or equal to 12 s.

Assessment of ventricular and atrial function and analysis of left-ventricular global strain were performed using the CVI42® software package (Circle Cardiovascular Imaging Inc., Calgary, Canada, Release 5.12.1). The mitral annulus plain systolic excursion (MAPSE) and the tricuspid annular plane systolic excursion (TAPSE) were calculated based on the difference between end-diastolic and end-systolic length measured at the lateral mitral annulus-to-apex and tricuspid annulus-to-apex, respectively, in the 4-chamber view.

### Quantitative blood-flow measurements by phase-contrast CMR

Free-breathing flow-sensitive phase-contrast pulse sequences (PC-CMR) were used to obtain transmitral and right upper pulmonary venous (RUPV) time-to-velocity blood-flow profile curves. To capture the fine structure of the blood-profile curves, a high temporal resolution of 10 ms was set. The following pulse-sequence parameters were applied: TR/TE/flip angle of 5 ms/3.3 ms/30°, a fixed velocity-encoding value of 100 cm/s was used for transmitral flow and 70 cm/s for the pulmonary vein. To speed up sequence acquisition, a parallel-imaging factor of two using SENSE was chosen. A variable number of heart phases based on the subject’s heart rate (i.e., heart rate of 60 bpm corresponds to 100 heart phases) was chosen. Blood flow profiles were evaluated using an analysis program (“HDZ MR-Tools” software package) developed at our institution [21].

**Fig. 1:**
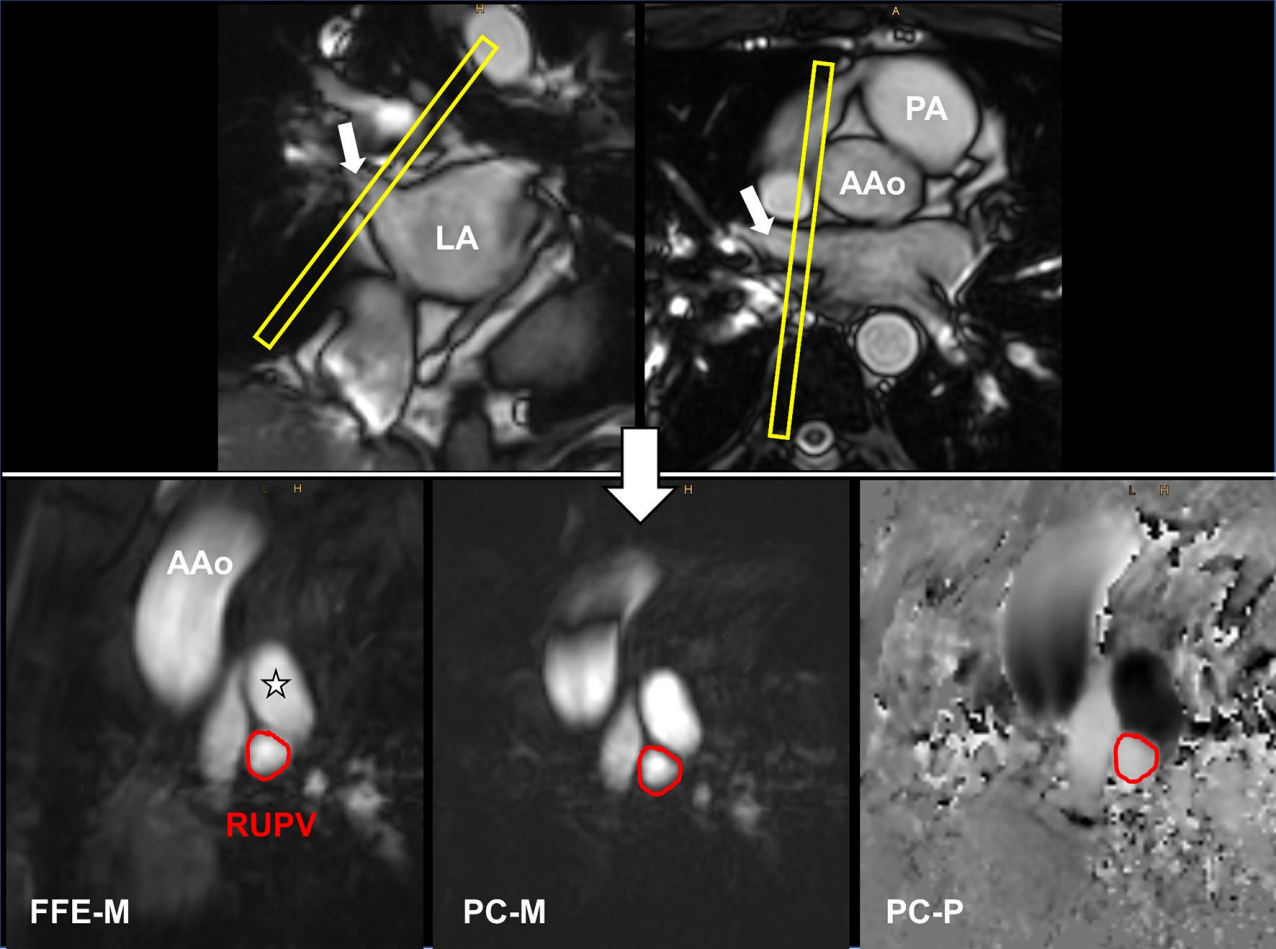
Planning (yellow bar) of the right upper pulmonary vein (RUPV) blood flow measurements. Upper left = coronal survey, upper right = axial cine SSFP. Lower left = magnitude image (FFE-M), lower middle = magnitude phase (PC-M) and lower right = phase image (PC-P) containing the blood flow information. *LA* left atrium, *AAo* ascending aorta, *PA* pulmonary artery

**Fig. 2:**
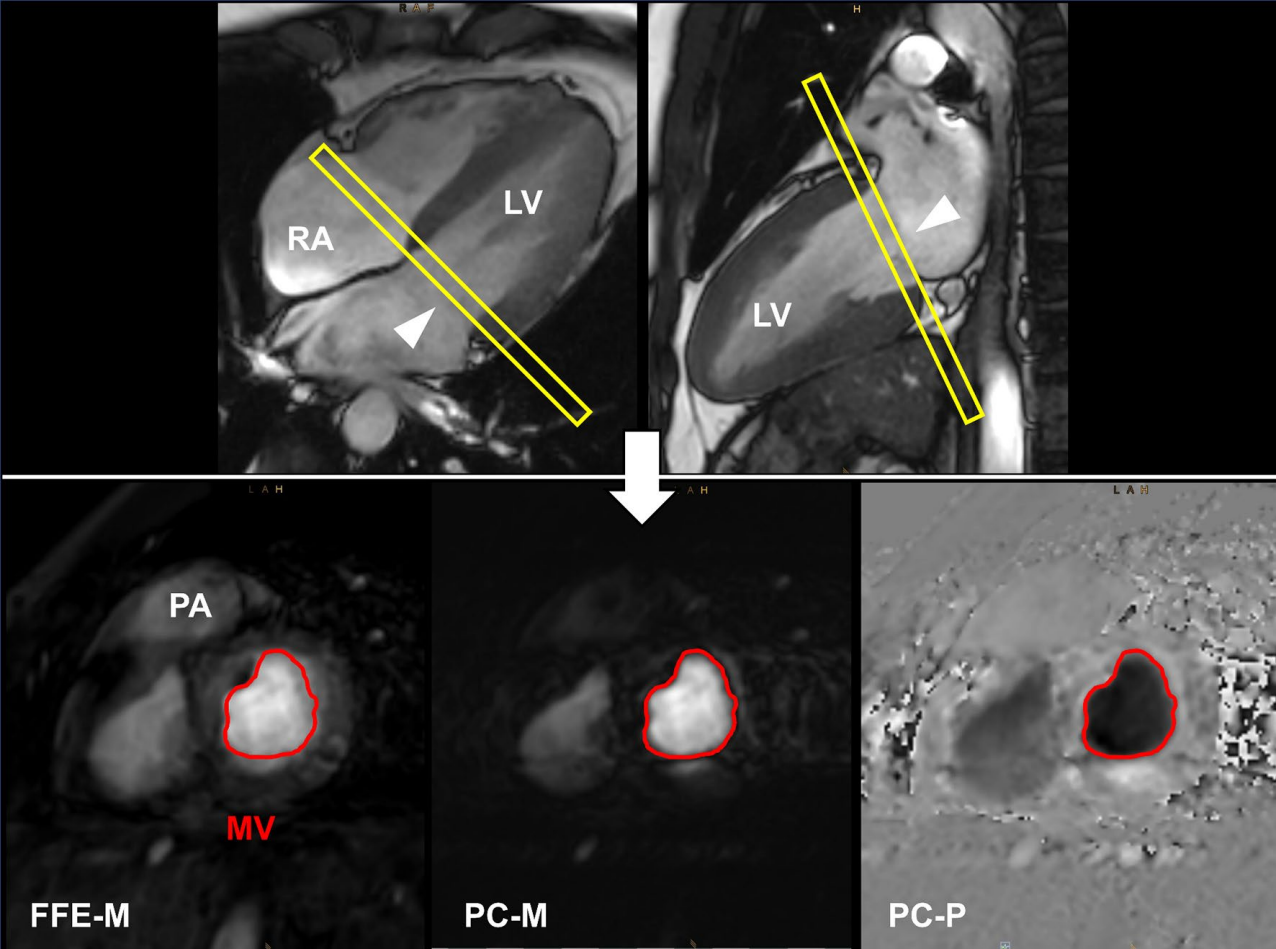
Planning (yellow bar) of the transmitral blood flow measurements. Upper left = end-systolic 4-chamber view, upper right = end-systolic 2-chamber view of a cine SSFP acquisition. Lower left = magnitude image (FFE-M), lower middle = magnitude phase (PC-M) and lower right = phase image (PC-P) illustrating the opening area of the transmitral blood flow measurement. *RA* right atrium, *LV* left ventricle, *PA* pulmonary artery

Accurate cross-sectional planning of the RUPV PC-CMR measurement was achieved using both a coronal and an axial cine SSFP acquisition as illustrated in Fig. 1. Accordingly, optimal planning of transmitral PC-CMR measurement was obtained using an end-systolic (just before mitral valve opening) 4-chamber view and an end-systolic 2-chamber view. The measurement position was placed at the top of the valve leaflets, as demonstrated in Fig. 2.

### Pulmonary vein and transmitral blood-flow analysis

**Fig. 3:**
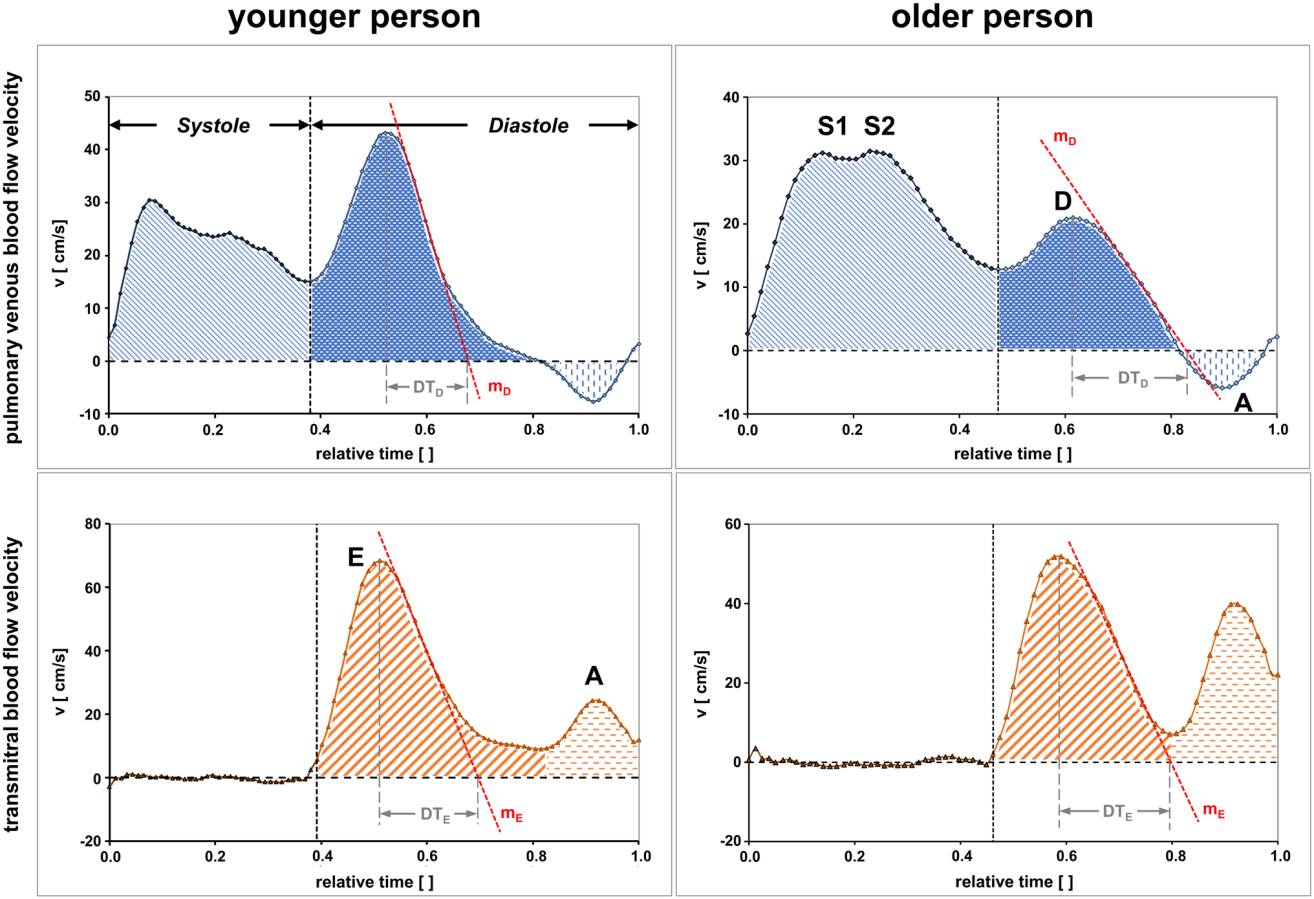
Evaluation of pulmonary venous (upper row) and transmitral (lower row) blood flow profiles. The different shadings indicate the different integrals. *S1 and S2* pulmonary venous systolic S1 and S2-wave; *D* pulmonary venous diastolic D-wave, *E* transmitral early diastolic E-wave; *A* transmitral late diastolic A-wave, *m*_*D*_ PV slope D-wave, *m*_*E*_ MV slope E-wave, *DT*_*D*_ PV deceleration time, *DT*_*E*_ MV deceleration time, *v* velocity

After transferring the image data from the PC-CMR measurements, the flow data were analyzed offline on an external workstation using a computer algorithm for semi-automatic detection of vessel boundaries. If needed, region-of-interests (ROI) were manually corrected in the magnitude images as usual by the operator. In addition to the time-to-velocity and time-to-volume curves, the software automatically detects the systolic peak values S1 and S2, the diastolic peak value D as well as the atrial reversal negative peak flow value A. Integrals were defined as shown in Fig. 3. The diastolic deceleration time (DT_D_) and the corresponding slope (*m*_D_) of the PV blood flow were calculated as well.

Accordingly, transmitral blood-flow analysis encompasses the detection of the peak diastolic passive emptying phase (E-wave), the peak diastolic active emptying phase (A-wave), the corresponding integrals, the deceleration time (DT_E_) and the corresponding slope (*m*_E_) as shown in Fig. 3. Because of the lack of boundaries, the ROI definitions for the transmitral blood-flow measurements were made manually in the magnitude phase images. This image type accurately depicts the blood flow as hyperintense areas.

The pulmonary capillary wedge pressure (PCWP) was calculated according to Nagueh et al. [19] using the formula PCWP = 1.9 ± 1.24*E/é, where é represents the early diastolic velocity of the mitral annulus, which was determined in the high temporal resolution CMR 4-chamber cine SSFP acquisitions. To achieve this goal, the opening of the mitral valve was first visually identified. The time stamp of this DICOM image was identified and the distances between the lateral and septal valve insertion points to the apex were determined. The same procedure was carried out two heart frames later. The spatial distances between the two measurement points on the lateral and septal sides were then calculated and, accordingly, the temporal difference between the two DICOM images. The lateral or septal é velocity in cm/secs was determined by the ratio of spatial and temporal difference. The mean value é derived from both measurements was then used in the Nagueh formula to estimate the PCWP.

### Echocardiography

As echocardiography is the gold standard for determining diastolic function, a subset of one hundred participants were examined immediately before or after the CMR examination on the same day for comparison purposes. For this purpose, a Vivid E9 (GE Medical Systems, Milwaukee, WI, USA, SCh, for speckle tracking) with standard probes was used. For data collection, the patients were examined according to a standard protocol. All echocardiographic examinations were performed by a single experienced specialist blinded to the CMR results. Accordingly, all echocardiographic results were blinded to the CMR expert.

### Statistics

SPSS (version 29.0, IBM Deutschland GmbH) was used for statistical analysis. The Shapiro–Wilk test was applied to test the normal distribution. Continuous variables are presented as mean ± standard deviation (SD) if normally distributed, otherwise as median with interquartile range. Gender differences were analyzed with the Mann–Whitney *U*-test in case of non-normally distributed data or with the unpaired Student’s t test if the data were normally distributed. A paired Student’s *t* test or the Wilcoxon-test was used to compare CMR data with echocardiographic data. Agreement between both measurements was assessed by Bland–Altman plots. Correlations between CMR data with echocardiographic data, PV measurements and age and between transmitral blood-flow measurements and age were calculated using Spearman or Pearson correlation, depending on the appropriate prerequisites such as linearity, normal distribution and the presence of outliers [22]. For all tests, a p value < 0.05 was considered as statistically significant. The correlation coefficient *r* was interpreted according to Cohen [23]. A strong correlation was assumed for r values above 0.5, a medium/moderate correlation for r values between 0.3 and 0.5 and a low/weak correlation for values between 0.1 and 0.3. The intra- and interobserver variability was analyzed using the intraclass correlation coefficient (ICC) and Bland–Altman statistics.

## Results

### Baseline characteristics

This study population consisted of 183 healthy participants with a median age of 34.2 years (interquartile range, IQR, 21.8–50.4 years). The median age of females and males was comparable (*p* = 0.409), whereas males demonstrated significantly increased weight, height, body surface area and body mass index (*p* < 0.001; Table 1).

**Table 1:** Baseline patient characteristics

	All	Male	Female	*p* value	Subgroup CMR vs. Echocardiography
n	183	86	97		100
Age (years)	34.2 {21.8; 50.4}	33.2 {21.1; 50.9}	34.5 {22.5; 49.3}	0.409	36 {23; 52}
Weight (kg)	69 {60; 81}	81 ± 15	61 {57; 68}	< 0.001^a^	70.0 ± 15
Height (cm)	173 ± 11	181 ± 8	166 ± 7	< 0.001	172 ± 11
Body surface area (m^2^)	1.85 ± 0.24	2.01 ± 0.21	1.69 ± 0.15	< 0.001^a^	1.82 ± 0.24
Body mass index (kg/m^2^)	23.2 {20.9; 26.0}	24.6 ± 3.7	22.5 {20.5; 24.5}	< 0.001^a^	23.0 {20.5; 25.9}

Data reported as mean ± standard deviation or median {interquartile range}

^a^ Mann–Whitney-*U*-test otherwise unpaired Student’s *t* test; *n* number of subjects. *p* values represent the statistical significance between males and females

Indexed left ventricular, right ventricular and left atrial end-diastolic and end-systolic volumes as well as the LV remodeling index were significantly greater for males compared to females (all *p* ≤ 0.004). In contrast, females presented increased biventricular ejection fractions and global left ventricular strain values for longitudinal, circumferential and radial cardiac deformation as well as higher LA passive emptying fractions than males (*p* < 0.001, Table 2). No gender-specific differences were found for the isovolumetric relaxation time (IVRT), mitral annular plane systolic excursion (MAPSE) and TAPSE.

**Table 2:** Ventricular and atrial cardiac function and left ventricular strain parameters measured by CMR

	All	Male	Female	*p* value
LV EDV_i_ (ml/m^2^)	75.5 ± 9.6	79.5 ± 8.7	72.0 ± 8.9	< 0.001^a^
LV ESV_i_ (ml/m^2^)	26.2 ± 5.5	28.4 ± 5.4	24.2 ± 4.9	< 0.001
LV SV_i_ (ml/m^2^)	49.4 ± 6.6	51.1 ± 6.5	47.8 ± 6.3	0.001^a^
LV ejection fraction (%)	65 ± 5	64 ± 5	67 ± 5	0.001
LV muscle mass_i_ (g/m^2^)	56.2 {49.5; 64.3}	64.0 ± 7.6	50.0 {44.5; 56.0}	< 0.001
LV GLS (%)	−16.9 ± 1.7	−16.0 ± 1.5	−17.6 ± 1.6	< 0.001
LV GCS (%)	−19.2 ± 2.1	−18.2 ± 1.8	−20.1 ± 1.8	< 0.001
LV GRS (%)	33.5 {29.6; 37.8}	31.2 ± 4.9	36.6 {32.8; 39.6}	< 0.001^a^
LA EDV_i_ (ml/m^2^)	46.9 {41.5; 54.1}	50.5 ± 9.8	45.4 {39.7; 51.7}	0.001
LA ESV_i_ (ml/m^2^)	20.7 {17.8; 25.1}	22.0 {18.9; 27.1}	20.1 {17.4; 23.5}	0.004^a^
LA SV_i_ (ml/m^2^)	26.4 {22.8; 29.8}	27.3 ± 4.7	25.8 {21.6; 29.0}	0.011
LA ejection fraction (%)	55 ± 5	55 ± 6	56 ± 5	0.070
RV EDV_i_ (ml/m^2^)	79.7 ± 12.2	86.0 ± 9.6	74.2 ± 11.5	< 0.001^a^
RV ESV_i_ (ml/m^2^)	31.0 {26.2; 37.0}	35.6 ± 6.7	27.5 {23.8; 31.6}	0.001^a^
RV SV_i_ (ml/m^2^)	47.9 ± 7.1	50.5 ± 6.6	45.5 ± 6.7	< 0.001
RV ejection fraction (%)	60 {56; 64}	58 {55; 62}	63 {57; 65}	< 0.001^a^
Mitral regurgitation (ml)	4.8 {−0.5; 9.1}	5.5 ± 9.3	4.5 {−0.9; 7.1}	0.097^a^
Tricuspid regurgitation (ml)	−2.9 ± 9.9	−0.4 ± 11.4	−5.2 ± 7.7	0.001
Isovolumetric relaxation time (ms)	82 {71; 99}	87 ± 26	84 ± 19	0.639
MAPSE (cm)	1.7 ± 0.3	1.7 ± 0.3	1.6 ± 0.3	0.148
TAPSE (cm)	2.2 {2.0; 2.5}	2.3 ± 0.4	2.3 {2.0; 2.5}	0.695
LV remodeling index (g/ml)	0.74 {0.67; 0.83}	0.80 {0.74; 0.86}	0.69 {0.64; 0.75}	< 0.001^a^
LA passive emptying fraction (%)	56.4 ± 11.8	53.6 ± 10.8	58.8 ± 12.1	0.003
LA active emptying fraction (%)	43.6 ± 11.8	46.4 ± 10.8	41.2 ± 12.1	0.003

Data reported as mean ± standard deviation or median {interquartile range}

*LV* left ventricle, *EDV* end-diastolic volume, *ESV* end-systolic volume, *SV* stroke volume, *GLS* global longitudinal strain, *GCS* global circumferential strain, *GRS* global radial strain, *LA* left atrium, *RV* right ventricle, *MAPSE* mitral annulus plain systolic excursion, *TAPSE* tricuspid annulus plain systolic excursion

^a^ Mann–Whitney-*U*-test otherwise unpaired Student’s *t* test; *p* values represent the statistical significance between males and females

### Gender dependency of the diastolic function

#### Pulmonal venous blood-flow pattern

Mean heart rate was 67 bpm (IQR 60;74) and comparable between both genders (*p* = 0.364). The gender-specific *blood-flow* characteristics by right upper pulmonary vein quantitative flow measurement are summarized in Table 3.

**Table 3:** Gender-specific blood-flow characteristics by right upper pulmonary vein quantitative flow measurement measured by CMR for describing diastolic function

	All	Male	Female	*p* value
Heart rate (bpm)	67 {60; 74}	67 ± 10	68 {61; 73}	0.364
PV S_max_-wave (cm/s)	30.5 {25.4; 37.9}	33.6 ± 9.5	29.4 {24.4; 35.4}	0.013
PV D-wave (cm/s)	28 {22.5; 33.7}	29.9 {24.3; 36.7}	27.4 ± 6.7	0.002
PV A-wave (cm/s)	−4.8 {−7.7; −2.4}	−4.5 ± 5.3	−4.4 {−7.8; −2.4}	0.873^a^
PV S/D (units)	1.1 {0.9; 1.4}	1.2 ± 0.4	1.1 {0.9; 1.4}	0.468
PV S/A (units)	5.2 {3.0; 8.6}	4.4 {2.5; 7.3}	6.0 {3.1; 9.1}	0.023^a^
PV D/A (units)	4.5 {2.8; 7.8}	4.3 {2.2; 6.6}	4.6 {3.1; 8.2}	0.041^a^
PV S integral (cm)	8.2 {6.4; 10.2}	8.8 ± 2.7	8.0 {6.3; 9.6}	0.061
PV D integral (cm)	5.6 {4.5; 7.0}	6.3 ± 1.7	5.2 {4.4; 6.6}	< 0.001
PV A integral (cm)	−0.3 {−0.6; −0.1}	−0.4 {−0.6; -0.1}	−0.3 {−0.6; −0.1}	0.949^a^
PV S/D integral (units)	1.45 {1.04; 1.89}	1.4 {1.0; 1.8}	1.5 {1.1; 1.9}	0.389^a^
PV S/A integral (units)	18.2 {7.2; 37.1}	14.6 {5.8; 30.8}	21.8 {8.9; 42.2}	0.019^a^
PV D/A integral (units)	11.7 {6.2; 25.7}	10.3 {4.8; 21.3}	12.4 {7.2; 27.1}	0.066^a^
PV mean velocity (cm/s)	15.7 ± 4.2	16.5 ± 4.6	15.1 ± 3.8	0.026^a^
PV stroke volume (ml)	23.9 {18.8; 30.1}	26.8 {22.5; 35.2}	20.5 {17.2; 25.9}	< 0.001^a^
PV slope D-wave (cm/s^2^)	-162 {−228; −117}	−172 {−238; −119}	−158 (−224; −114)	0.300^a^
PV deceleration time (ms)	183 {156; 224}	188 {157; 225}	181 {154; 220}	0.636^a^

Data reported as mean ± standard deviation or median {interquartile range}

*PV* pulmonary veins, *S/D ratio* S-wave to D-wave, *S/A ratio* S-wave to A-wave, *D/A ratio* D-wave to A-wave

^a^ Mann–Whitney-*U*-test otherwise unpaired Student’s t test; P values represent the statistical significance between males and females

No gender-specific differences are found for the most established measures describing diastolic function, such as PV S/D, PV S/D integral, PV slope D-wave or PV deceleration time, although velocity values (PV *S*_max_-wave, PV D-wave) are significantly increased for males in contrast to females (PV *S*_max_-wave: 33.6 ± 9.5 cm/s vs. 29.4 cm/s (IQR 24.4–35.4; *p* = 0.013); PV D-wave: 29.9 cm/s (IQR 24.3–36.7) vs. 27.4 ± 6.7 cm/s; *p* = 0.002).

#### Transmitral blood-flow pattern

The heart rates in the transmitral (MV) *blood-flow* measurements did not differ between the two sexes (mean heart rate of 66 bpm (IQR 60 to 74 bpm), Table 4).

**Table 4:** Gender-specific blood-flow characteristics by transmitral quantitative flow measurements measured by CMR

	All	Male	Female	*p* value
Heart rate (bpm)	66 {60; 74}	67 ± 9	68 {61; 74}	0.127
MV E-wave (cm/s)	52.7 ± 9.1	51.1 ± 9.1	54.1 ± 9.0	0.007^a^
MV A-wave (cm/s)	28.3 {22.7; 33.9}	27.4 {22.3; 34.0}	29.0 ± 7.9	0.493
MV E/A (units)	1.9 {1.6; 2.4}	1.9 ± 0.6	1.9 {1.6; 2.4}	0.452^a^
MV E integral (cm)	9.4 ± 1.8	9.4 ± 1.8	9.4 ± 1.9	0.493
MV A integral (cm)	3.4 {2.8; 4.2}	3.7 ± 1.0	3.5 ± 0.9	0.085
MV E/A integral (units)	2.7 {2.2; 3.4}	2.6 {2.0; 3.5}	2.8 {2.2; 3.3}	0.504^a^
MV mean velocity (units)	14.8 {13.0; 16.6}	14.7 {12.8; 16.6}	14.9 ± 2.4	0.460
MV stroke volume (ml)	94.9 ± 20.9	108.5 ± 17.6	82.8 ± 15.6	< 0.001
MV slope E-wave (cm/s^2^)	−302 {−359; −260}	−292 {−338; −243}	−319 ± 82	0.047^a^
MV deceleration time (ms)	183 {170; 207}	189 {172; 211}	181 {168; 206}	0.156^a^
MV mean é (cm/s)	13.3 ± 4.0	13.3 ± 4.2	13.2 ± 3.8	0.455
MV E/é (units)	4.1 {3.4; 5.0}	4.0 {3.3; 4.8}	4.2 {3.7; 5.1}	0.128^a^
PCWP (mmHg)	7.1 {6.2; 8.1}	6.8 {6.0; 7.8}	7.1 {6.4; 8.2}	0.127^a^

Data reported as mean ± standard deviation or median {interquartile range}

*MV* mitral valve, *E/A ratio* E-wave to A-wave, *é* mitral annulus early diastolic CMR velocity, *E/é ratio* E-wave to é, *PCWP* pulmonary capillary wedge pressure

^a^ Mann–Whitney-U-test otherwise unpaired Student’s t test; *p* values represent the statistical significance between males and females

Similar to the RUPV *blood-flow* measurements, for the most relevant measures describing diastolic function, such as MV E/A, MV E/A integral, MV deceleration time and MV E/é, no gender-specific differences were detected, except for the slightly lower MV slope of the E-wave in males compared to females (−292 cm/s^2^ {IQR −338 to −243 cm/s^2^} vs. −319 ± 82 cm/s^2^, *p* = 0.047). In regard to the absolute values, only the MV E-wave was statistically lower for males than for females (51.1 ± 9.1 cm/s vs. 54.1 ± 9.0 cm/s, *p* = 0.007).

### Age dependency of the diastolic function

#### Pulmonal venous blood-flow pattern

**Fig. 4:**
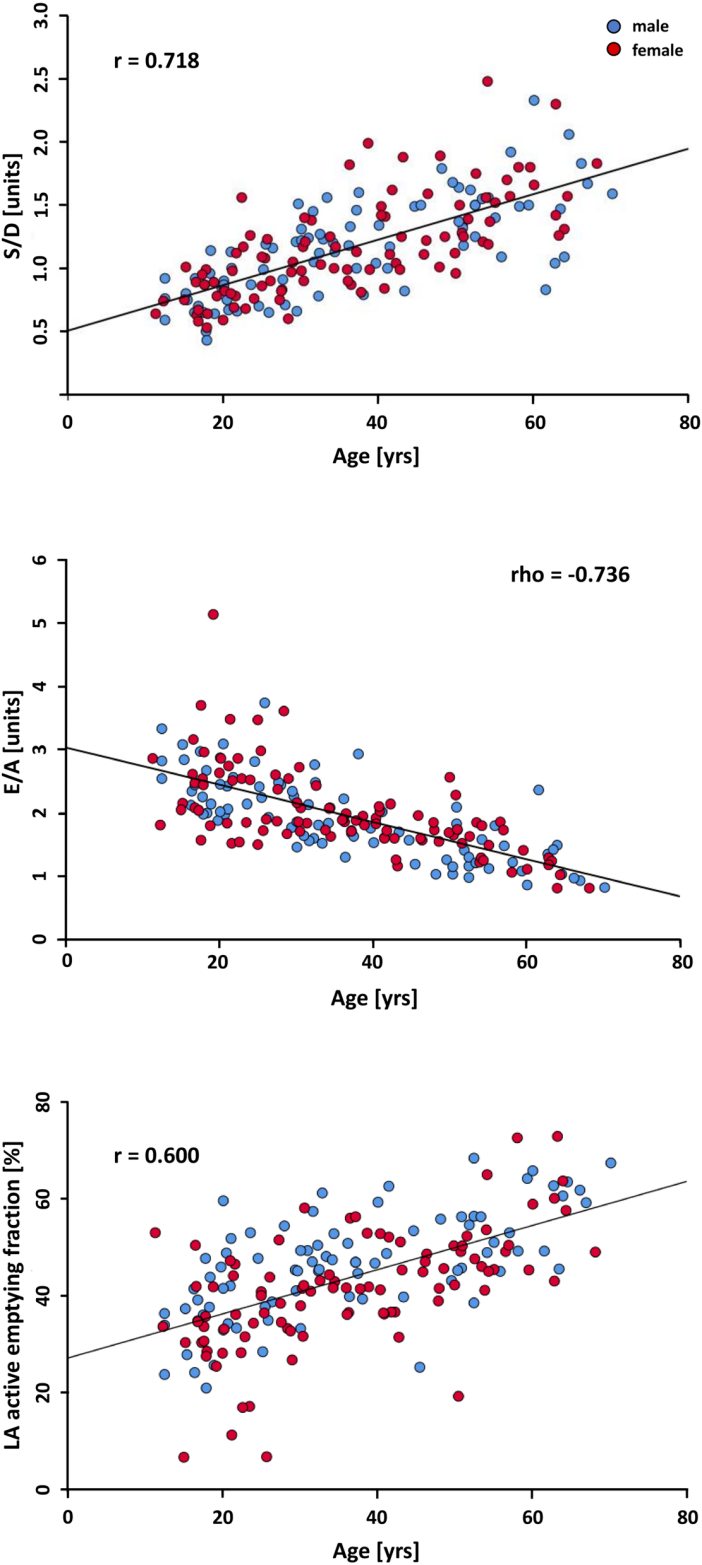
Linear regression analysis of age-dependent change of pulmonary venous and transmitral diastolic function parameters, measured using CMR. The pulmonary venous S/D ratio is shown above; the transmitral E/A ratio is depicted in the center and the LA-active emptying fraction is presented below. *r* Pearson product–moment correlation, *rho* Spearman’s Rho correlation

The correlations between PV *blood-flow* characteristics of the total cohort and age are given in Table 5. Almost all PV measures describing diastolic function correlate statistically significantly with age, with the exception of the PV A-wave and its associated measures. In particular, the PV S/D ratio correlates strongly with age according to Cohen et al. [23] with a value of *r* = 0.718 (*p* < 0.001), as is also the case for the ratio of PV S/D integral (*r* = 0.662, *p* < 0.001). The positive correlation indicates that the diastolic PV D-wave portion decreases with increasing age, relative to the PV S-wave portion. This is underlined by the strong negative correlation (*r* = −0.545, *p* < 0.001) of PV D-wave, indicating that the D-wave decreases with increasing age, alongside a moderate positive correlation (*r* = 0.328, *p* < 0.001) of the PV S-wave, reflecting an increase with increasing age (see Figs. 3 and 4). Furthermore, the moderately positive correlation of the PV slope D-wave (*r* = 0.394, *p* < 0.001) demonstrates that the D-wave increasingly flattens with age, as indicated by the negative algebraic sign of this parameter (see Fig. 3).

**Table 5:** Correlations between pulmonary venous blood-flow characteristics of the total cohort and age

	r resp. rho	P value
PV S_max_-wave (cm/s)	0.328	< 0.001
PV D-wave (cm/s)	**−0.545**	< 0.001
PV A-wave (cm/s)	−0.069	0.354^a^
PV S/D (units)	**0.718**	< 0.001
PV S/A (units)	0.210	0.004^a^
PV D/A (units)	−0.083	0.267^a^
PV S integral (cm)	0.444	< 0.001
PV D integral (cm)	−0.410	< 0.001
PV A integral (cm)	−0.014	0.849^a^
PV S/D integral (units)	**0.662**	< 0.001
PV S/A integral (units)	0.238	0.001^a^
PV D/A integral (units)	0.029	0.696^a^
PV mean velocity (cm/s)	0.007	0.924
PV stroke volume (ml)	0.479	< 0.001
PV slope D-wave (cm/s^2^)	0.394	< 0.001
PV deceleration time (ms)	0.176	0.017^a^

Interpretation according to Cohen [23], weak correlation = 0.10, moderate correlation = 0.30, strong correlation = 0.50 (**bold**)

*PV* pulmonary veins, *S/D ratio* S-wave to D-wave, *S/A ratio* S-wave to A-wave, *D/A ratio* D-wave to A-wave

^a^ Spearman’s Rho correlation otherwise Pearson product–moment correlation

#### Transmitral blood-flow pattern

As shown in Table 6, with the exception of the MV deceleration time, all transmitral measures describing diastolic function correlate statistically significantly with increasing age (total cohort). The strong negative correlations of the MV E/A ratio (*ρ* = −0.736, *p* < 0.001) and the MV E/A integral ratio (*r* = −0.746, p < 0.001), respectively, indicate that the MV E-wave portion decreases with increasing age, while the MV A-wave portion rises. This behaviour is confirmed by the negative correlation of the MV E-wave with age (*r* = −0.472, *p* < 0.001, E-wave reduction) and by the strong positive correlation of the MV A-wave with age (*r* = 0.598, *p* < 0.001, A-wave increase). In addition, the weak positive correlation of the MV slope E-wave (*ρ* = 0.235, *p* < 0.001) indicates that the E-wave flattens with increasing age as the values of this parameter have a negative algebraic sign as well (see Fig. 3 and 4). Although strictly it is not a transmitral measure, the study reveals that the active LA-emptying fraction grows with increasing age of the participants (*r* = 0.600, *p* < 0.001).

**Table 6:** Correlations between various parameters of transmitral blood flow and related measures of the total cohort and age

	r resp. rho	*p* value
MV E-wave (cm/s)	−0.472	< 0.001
MV A-wave (cm/s)	**0.598**	< 0.001
MV E/A (units)	−**0.736**	< 0.001^a^
MV E integral (cm)	−0.486	< 0.001
MV A integral (cm)	**0.632**	< 0.001
MV E/A integral (units)	−**0.746**	< 0.001^a^
MV mean velocity (units)	−0.310	< 0.001
MV stroke volume (ml)	0.031	0.672
MV slope E-wave (cm/s^2^)	0.235	0.001^a^
MV deceleration time (ms)	−0.042	0.572^a^
MV mean é (cm/s)	−0.490	< 0.001
MV E/é (units)	0.248	< 0.001^a^
PCWP (mmHg)	0.244	< 0.001^a^
LV remodeling index (g/ml)	0.342	< 0.001
LA passive emptying fraction (%)	**-0.600**	< 0.001
LA active emptying fraction (%)	**0.600**	< 0.001

Interpretation according to Cohen [23], weak correlation = 0.10, moderate correlation = 0.30, strong correlation = 0.50 (**bold**)

*MV* mitral valve, *PCWP* pulmonary capillary wedge pressure, *LV* left ventricle, *LA* left atrium, *E/A ratio* E-wave to A-wave, *é* mean lateral and septal cine CMR velocities, *E/é ratio* E-wave to é

^a^ Spearman’s Rho correlation otherwise Pearson product–moment correlation

#### Generalization of the different behavior of younger persons compared to older persons

Due to the higher rate of cardiovascular disease in the second half of life, it is common to report the average values of diastolic parameters during this period [24]. The recommendations for the evaluation of left ventricular diastolic function by echocardiography published in the ASE/EAVI guidelines are also based on such average values and serve to define an algorithm for the diagnosis of diastolic dysfunction [3]. As adolescence and young adults (up to 30 years of age) are not adequately considered in these guidelines, a distinction between healthy younger individuals and healthy older individuals is proposed in this study.

**Fig. 5:**
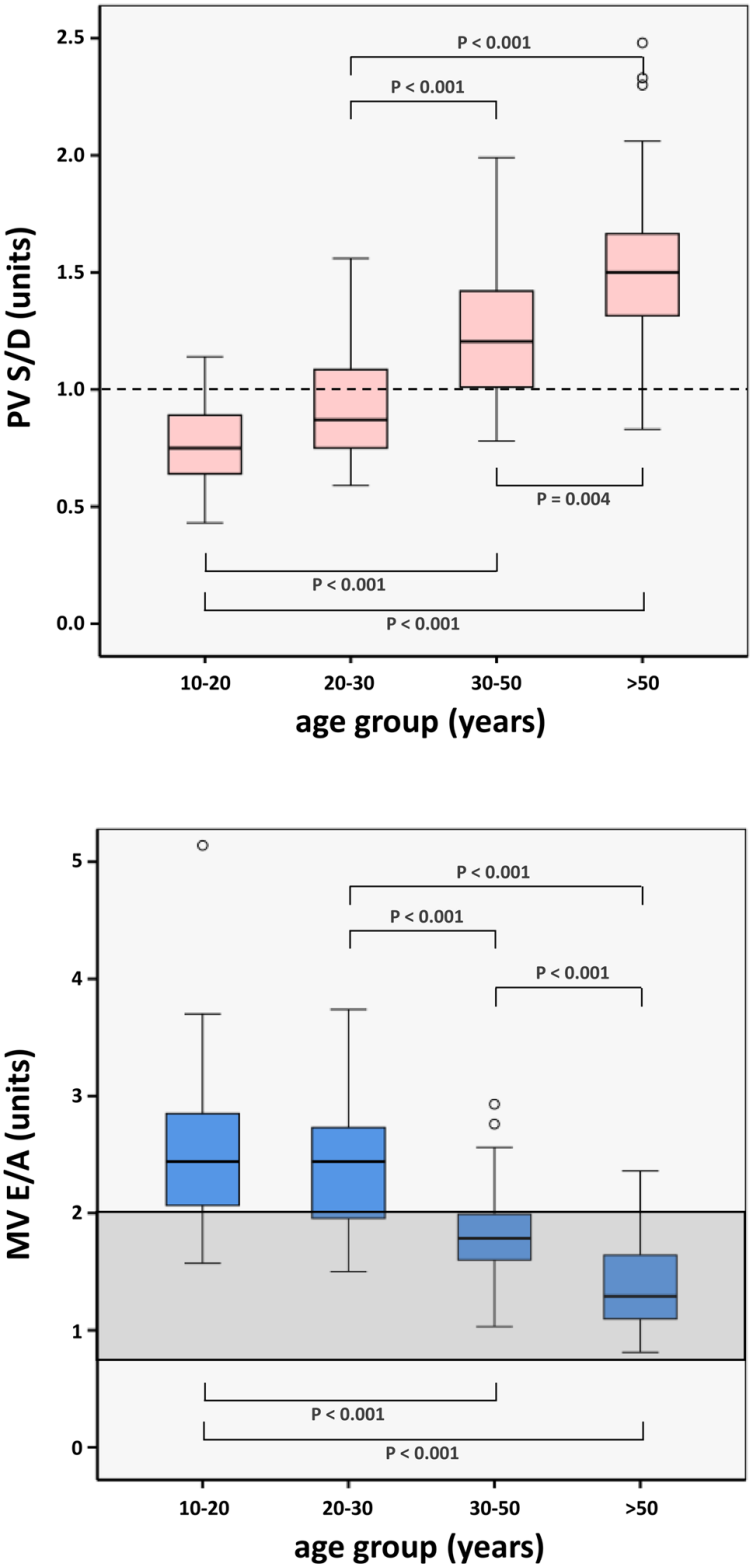
Boxplots of the pulmonary venous (PV) S/D ratio and transmitral (MV) E/A ratio, classified by different healthy age groups. The dashed line for the PV S/D ratio and the gray-shaded area for the MV E/A ratio represents the normal (healthy) stage 0 according to references [32, 35]

While no differences were found in the PV S/D or MV E/A ratios between the two decades 10–20 years (group A) vs. 20–30 years (group B), the individuals ≥ 30-years (group C) differed statistically significantly from the other two study groups (all *p* < 0.001, see Table 7, Fig. 5).

**Table 7:** Different behavior of younger individuals compared to older individuals in relation to diastolic function. Age-subgroups of some key parameters assessed by CMR quantitative flow measurements in the right upper pulmonary vein and through the mitral valve

	A (10–20 yrs)	B (20–30 yrs)	C (30–50 yrs)	D (> 50 yrs)	Comparison	Post-hoc test^a^
N	31	43	62	47		
PV S/D (units)	0.76 ± 0.17	0.87 {0.75; 1.09}	1.21 {1.01; 1.43}	1.50 {1.31; 1.67}	A–B A–C A–D B–C B–D C–D	*p* = 0.270 *p* < 0.001 *p* < 0.001 *p* < 0.001 *p* < 0.001 *p* = 0.004
PV S/D integral (units)	1.00 ± 0.29	1.06 {0.90; 1.47}	1.54 {1.26; 1.83}	2.05 ± 0.51	A–B A–C A–D B–C B–D C–D	*p* = 0.623 *p* < 0.001 *p* < 0.001 *p* < 0.001 *p* < 0.001 *p* = 0.004
MV E/A (units)	2.44 {2.05; 2.86}	2.42 ± 0.56	1.79 {1.60; 2.00}	1.38 ± 0.38	A–B A–C A–D B–C B–D C–D	*p* = 1.000 *p* < 0.001 *p* < 0.001 *p* < 0.001 *p* < 0.001 *p* < 0.001
MV E/A integral (units)	3.63 {2.95; 4.31}	3.61 {2.93; 4.29}	2.65 ± 0.53	1.91 ± 0.44	A–B A–C A–D B–C B–D C–D	*p* = 1.000 *p* < 0.001 *p* < 0.001 *p* < 0.001 *p* < 0.001 *p* < 0.001

For better comparison purposes with echocardiographic data, the ratios PV S/D and MV E/A should be used

Data reported as mean ± standard deviation or median {interquartile range}

*MV* mitral valve, *PV* pulmonary vein

^a^ Kruskal–Wallis-Test as global test with Mann–Whitney *U*-test for pairwise comparisons with Bonferroni correction

### Comparison between echocardiography and CMR data

In a subgroup of 100 study participants, echocardiographic data were compared with CMR data. Blood flow characteristics and correlations of the two modalities are shown in Table 8. With the exception of the PV S/D and MV E/A ratios, all absolute values measured by echocardiography were significantly higher than those determined by CMR.

**Table 8:** Pulmonary venous and transmitral blood-flow characteristics and correlations between CMR and echocardiography assessed in a subgroup of *N* = 100

	CMR	Echo	*P* value^c^	Correlation r resp. rho	Bland–Altman^e^
PV Heart rate (bpm)	67 ± 9	67 {60; 74}	0.320^b^		
PV S_max_-wave (cm/s)	30.6 {24.8; 36.8}	61.0 ± 13.8	< 0.001^b^	0.106	−29.0 (3.6; −61.5)
PV D-wave (cm/s)	27.3 {22.2; 32.1}	56.3 ± 13.7	< 0.001^b^	0.352	−28.4 (−2.8; −54.4)
PV A-wave (cm/s)	−4.9 {−7.7; −2.6}	−24.5 ± 5.7	< 0.001^a^	0.183^d^	19.6 (32.8; 6.4)
PV S/D (units)	1.15 {0.9; 1.5}	1.13 ± 0.3	0.100^b^	**0.545**	0.07 (0.78; −0.65)
PV S integral (cm)	8.4 ± 2.6	15.7 ± 4.2	< 0.001^b^	0.242	−7.3 (1.5; −16.0)
PV D integral (cm)	5.7 ± 1.6	11.8 {9.9; 14.1}	< 0.001^b^	0.313	−6.2 (0.1; −12.5)
PV S/D integral (units)	1.5 {1.1; 1.9}	1.3 {1.1; 1.6}	0.039^a^	**0.529**^d^	0.16 (1.27; −0.95)
PV deceleration time (ms)	183 {157; 222}	204 {173; 239}	0.013^a^	0.459^d^	−13 (111; −138)
MV Heart rate (bpm)	67 ± 9	66 {61; 73}	0.305^b^		
MV E-wave (cm/s)	51 ± 9.1	81.1 ± 17.7	< 0.001^b^	**0.685**	−30.0 (−3.6; −56.4)
MV A-wave (cm/s)	28.6 {22.0; 34.3}	58.9 ± 14.5	< 0.001^b^	**0.533**	−30.0 (−5.5; −54.5)
MV E /A (units)	1.9 ± 0.6	1.4 {1.1; 1.7}	< 0.001^a^	**0.692**^d^	0.45 (1.59; −0.69)
MV E integral (cm)	9.2 ± 1.9	13.4 ± 3.3	< 0.001^b^	**0.626**	−4.2 (0.86; −9.3)
MV A integral (cm)	3.6 ± 0.9	7.0 {5.8; 8.5}	< 0.001^a^	**0.542**^d^	−3.6 (0.32; −7.5)
MV E/A integral (units)	2.5 {2.0; 3.3}	2.0 {1.4; 2.4}	< 0.001^a^	**0.623**^d^	0.64 (2.58; −1.30)
MV deceleration time (ms)	187 ± 28	178 ± 43	0.041^b^	0.198	9 (101; −83)
MV E/é (units)	3.99 {3.1; 4.8}	5.3 {4.9; 6.7}	< 0.001^a^	0.337^d^	−1.7 (1.9; −5.2)
PCWP (mmHg)	6.8 {5.7; 7.8}	8.5 {7.9; 10.2}	< 0.001^a^	0.333^d^	−2.1 (2.3; −6.4)

Interpretation according to Cohen [23], weak correlation = 0.10, moderate correlation = 0.30, strong correlation = 0.50 (**bold**)

Data reported as mean ± standard deviation or median {interquartile range}

*PV* pulmonary veins, *S/D ratio* S-wave to D-wave, *MV* mitral valve, *PCWP* pulmonary capillary wedge pressure, *E/A ratio* E-wave to A-wave, *E/é ratio* E-wave to é

^a^ Mann–Whitney-*U*-test

^b^ unpaired Student’s *t* test

^c^ *p* values represent the statistical significance between CMR and echocardiography

^d^ Spearman’s Rho correlation otherwise Pearson product–moment correlation

^e^ Bland–Altman statistics representing the mean difference and the upper/lower limits-of-agreement

**Fig. 6:**
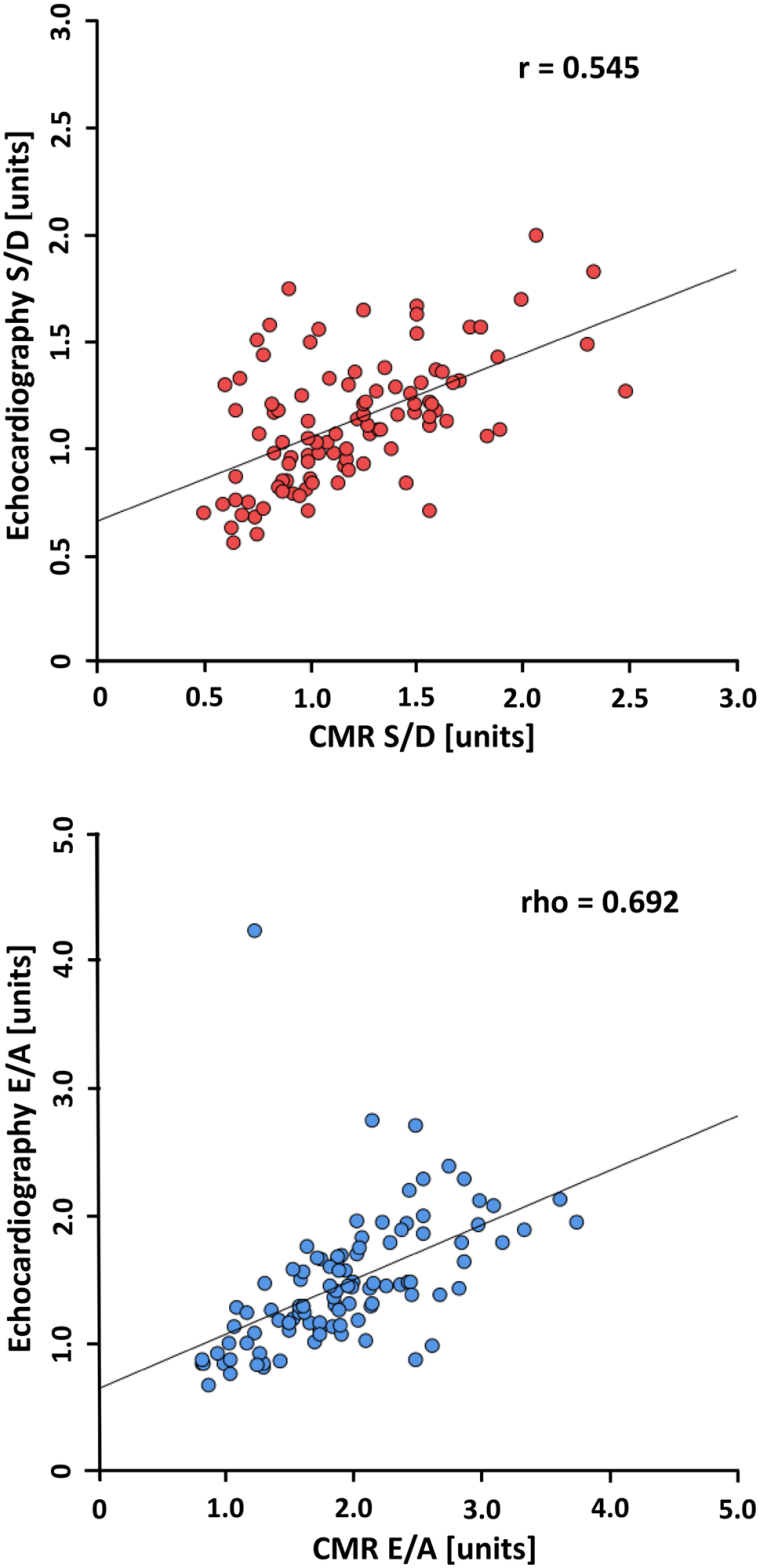
Comparison between CMR and echocardiography data. The pulmonary venous S/D ratio is shown in the upper row; the transmitral E/A ratio is depicted in the lower row. *r* Pearson product–moment correlation, *rho* Spearman’s Rho correlation

However, strong correlations were found for the PV *blood-flow* ratios PV S/D (*r* = 0.545, *p* < 0.001) and PV S/D integral (*r* = 0.529, *p* < 0.001) as well as for the two transmitral *blood-flow* ratios MV S/D (*r* = 0.692, *p* < 0.001) and MV S/D integral (*r* = 0.623, *p* < 0.001, see Fig. 6). A slightly lower correlation was observed for the PV deceleration time (*r* = 0.459, *p* < 0.001). Although statistically significant, only a weak correlation for the MV deceleration time (*r* = 0.198, *p* = 0.049) was observed between the two examination techniques.

### Intra- and interobserver

Inter- and intraobserver variability for CMR quantifications was evaluated in 20 study participants randomly selected from the total cohort. Bland–Altman plots and correlation diagrams can be found in the supplement. Intra- and interobserver variability of PV flow as calculated with the Bland–Altman statistics was < 4%, with the exception of the PV deceleration time, which was 7.8%. The correlation coefficients were all > 0.9, with the exception of the PV deceleration time and PV slope D-wave, which were around 0.7.

Intra- and interobserver variability of transmitral flow as calculated with the Bland–Altman statistics was slightly higher with values < 8%, with the exception of the interobserver MV E/A integral, which was 11.5%. All correlation coefficients were > 0.8.

## Discussion

This study currently represents the most extensive CMR investigation conducted on healthy individuals, aiming to provide reliable normal reference values for diastolic function parameters over the age range between 10 and 70 years and for both sexes. An attempt was made to distribute the participants evenly over all 6 decades to improve statistical accuracy. The diastolic function was characterized by examining the flow pattern of the right upper PV blood flow and the transmitral blood flow using flow-sensitive phase-contrast CMR. Biventricular and left atrial function as well as left ventricular strain were also assessed to obtain a comprehensive picture of the diastolic function.

The main novelties of this study are as follows:

i) CMR demonstrated significant gender-differences in absolute values of PV and transmitral flow, however their clinical impact appears subtle and is reflected in gender comparable functional ratios, per example by PV S/D and MV E/A.
ii) CMR characterizes significant age-dependent alterations in PV and MV hemodynamics, most notably evidenced by increasing PV S/D ratios and decreasing MV E/A ratios as age advances.
iii) CMR and echocardiographic acquired values differ significantly, implying high intermodal discrepancy and the need for separate CMR reference values.
iv) CMR intra- and interobserver variability analysis consolidates reliable levels of reproducibility, particularly regarding flow assessments.

In recent years, there have been a few studies in which diastolic dysfunction has been investigated using CMR. Typically, patient cohorts were only compared with a limited number of healthy individuals, so that a clear distinction from the pathologic state is limited [25–28]. Two studies have previously assessed the age dependence of diastolic function parameters using phase contrast CMR to provide reference values [12, 29]. However, the focus was mainly on mitral blood-flow parameters, while we considered both mitral and PV blood-flow parameters as well as the LA passive/active emptying fraction.

In particular, it seems that the influence of patient ageon diastolic function parameters was insufficiently considered in the 2016 ASE/EACVI guideline [3]. As underscored by Popovic et al., the ASE classification carries the risk of classifying people as sicker or healthier than they are. According to this classification, healthy individuals specifically under the age of 30, commonly showed signs of grade III diastolic dysfunction, as their E/A ratio was found > 2 [24]. To close this gap, the present study included not only young individuals, but also a broad age range.

### Gender dependency of the diastolic function

Although absolute velocity values such as PV S_max_-wave, PV D-wave or MV E-wave do show gender-differences, they are more related to known physiological gender-differences such as height, weight, body surface index or indexed LV stroke volumes (Tables 1 and 2). However, since the relevant parameters for describing the diastolic function are mainly based on ratios (PV S/D, MV E/A) or represent slopes or deceleration times, they remain independent of absolute velocity values or integrals. Consequently, the influence of gender appears to have a minor clinical impact. Therefore, all study participants were utilized to investigate the age-dependency.

### Age dependency of the diastolic function

This study assessed the age-dependent alterations of diastolic function that occur as a consequence of left atrial and left ventricular remodeling and compliance reduction with advancing age. Moreover, these observations emphasize the strong atrioventricular interdependence, that may translate into pathophysiological developments. CMR assessment determined significant increase in the PV S/D ratio and significant decrease in the MV E/A ratio as the age of healthy individuals increased.

In this study an S/D ratio of > 1 was found for the age-group > 30 years, which is in line with other studies [15, 30], whereas an S/D ratio < 1 was determined in the two younger groups. This is a very important issue and needs to be carefully considered as such ratios are typically only found in patients with moderate to severe reduced LV compliance [3, 15].

The assessment of the S/D ratio for the staging of diastolic dysfunction according to American Society of Echocardiography is limited in young people, as they often have an S/D ratio < 1. However, this was described as physiological in the study by Mandinov et al. [15]. Further points of criticism of the current grading for diastolic function were highlighted in the study by Buffle et al. [31] This study emphasized the diagnostic and prognostic clinical value of PV-flow parameters. Their research identified S/D integral ratio as the best predictor of heart failure readmission and the most effective diagnostic parameter for detecting early stage of diastolic dysfunction [12].

Moreover, age-related changes in the flow pattern were reflected by the flattening of the PV slope of the D wave in heart-healthy subjects (see Fig. 3). Hence, the PV slope D-wave could serve as an additional valuable metric for delineating the subclinical diastolic impairment of left ventricular compliance that manifests with age progression.

In healthy young people (< 30 years), early diastolic filling dominates the LV filling phase because of low LV diastolic pressure, increased compliance and rapid LV relaxation [3, 15, 32]. This explains the E/A ratio > 2 in the two younger study groups. Normally the E/A ratio is > 1, but an impaired ventricular relaxation and reduced ventricular filling can lead to E/A values < 1 [3, 15, 32]. However, even healthy people with normal diastolic function can have an E/A ratio < 1, for example with increasing age, as found in our data. However, this appears to be a physiological adaption, as stated by Schirmer et al. [33]. This can be explained by the fact that early diastolic filling is attenuated, while late diastolic filling, resulting from atrial contraction, increasingly provides the compensatory force for LV filling. This compensatory effect is further confirmed by the strong positive correlation between age and the LA-active emptying fraction displayed in this study.

### Comparison between echocardiography and CMR data

Strong correlations were observed between both imaging techniques for the most important measures describing diastolic function such as the PV S/D ratio and the E/A ratio, but also for most other diastolic function parameters. Buss et al. demonstrated even stronger correlations for the E/A ratio, examining however a heterogenous group of patients with various cardiovascular diseases combined with healthy volunteers [12]. Furthermore, it should be noted that the majority of PV and MV flow parameters presented significant intermodal differences as the absolute values of the velocity-based parameters of the PV and transmitral flow pattern were generally higher with echocardiography. This is consistent with other researchers demonstrating higher transmitral peak velocities with echocardiography compared to phase-contrast CMR [12, 25, 34]. A possible explanation to the observation could be that echocardiography imaging determines flow data in real-time, whereas CMR acquired flow data is typically averaged over a longer time interval of several seconds up to 2–3 min. This averaging prevents, to a certain amount, the instantaneous and accurate estimation of peak velocities and leads to a lowering of the velocity values when CMR is applied. The discrepancies underscore the need for modal-dependent reference values to prevent misclassification between diastolic function and dysfunction. Conversely, Ramos et al., found reduced absolute values for echocardiographic parameters in contrast to CMR acquired parameters [27]. Despite the high level of CMR reproducibility reflected in the inter- and intraobserver variability results, the normal values in this study require comparison with larger cohort data to establish age- and gender dependent reference values.

Other recommended variables for determining whether LV diastolic function is normal or abnormal are the mitral E/é ratio and the PCWP, which were also evaluated in this study. Although differences between the two imaging techniques were statistically significant, the values of both variables indicate that they are within the expected normal range [3].

### Limitations

The present study was conducted as a cross-sectional study at a single-center. Causality cannot be determined with the study design. The study is limited to a Caucasian cohort of healthy volunteers. Since fewer individuals in the > 50 age group meet the criteria for cardiac healthiness, the generalizability of the age-related results in this group may be somewhat limited. However, efforts were made to select optimal candidates within this age segment who met criteria for cardiac healthiness to mitigate potential bias.

To prevent different testing conditions and possible physiological variations between the CMR and echocardiographic examinations, all participants were assessed either immediately before or after completing the examination procedure of the other modality on the same day and in the same local unit. However, it should be mentioned that (1) CMR was conducted in the supine position, whereas for echocardiography a left supine position was used to obtain standard views, and (2) the study participants had to transition between examinations. The impact of these slightly different physiological conditions, such as an altered heart rate or variations in blood-flow characteristics, remains undetermined.

A key strength of this study is that it utilizes standard equipment, such as a clinical MR scanner, routine CMR pulse sequences, commercially available analysis software, to assess a large proportion of CMR parameters such as ventricular/atrial volumes, strains, LA-emptying fraction, PCWP and é. These parameters are feasibly assessed and consequently well suited for routine clinical use. The only exception is the description of the PV and transmitral blood-flow pattern to assess the PV slope D-wave, the MV slope E-wave and deceleration times, which required analysis on an external workstation. The inclusion of these parameters in clinical practice would necessitate Medical Device Regulation (MDR) approval. While this was not the aim of our study, it would be highly valuable, as both slopes showed a strong statistical correlation with patient age.

## Conclusion

CMR reliably measures diastolic parameters across a broad age spectrum of healthy individuals. Unlike gender, aging emerges as a pivotal factor influencing the development of the PV and MV blood-flow patterns. Findings correlate with echocardiography but with differences in absolute values emphasizing the need for tailored reference values for each modality. Hence, CMR can be considered a promising alternative to echocardiography for diastolic function analysis.

## Data Availability

The data underlying this article will be shared on reasonable request to the corresponding author.
